# Diseases of Children

**Published:** 1895-01-19

**Authors:** 


### MEDICAL PROGRESS AND HOSPITAL CLINICS.

[*The Editor will be glad to receive offers of co-operation and contributions from members of the profession. All letters should be addressed to* THE EDITOR, THE LODGE, PORCHESTER SQUARE, LONDON, W.]

### PROGRESS IN MEDICINE.

### DISEASES OF CHILDREN.

**Infantile Convulsions and Epilepsy.**—In a clinical lecture on this subject, Dr. Gowers[1] remarks that convulsions in rachitic infants are due to a retarded development of the system generally, and of the nervous structures more particularly. In a healthy infant the higher centres as they develop exercise a controlling power over the lower or reflex centres, but this does not hold good if rickets is present. The reflex irritation of dentition is therefore sufficient to cause the discharge from the cortical motor cells, which we term a convulsion, and when this has happened once there remains a liability ("residual influence") for it to happen again, or to break out at puberty as epilepsy, or to continue through life without any distinct period of freedom from the affection. The convulsions due to rickets are general in their distribution. As regards the treatment, he lays stress on the continued administration of bromides for some time after the cessation of the convulsions, so as to counteract the residual tendency referred to. This is of special importance in those cases in which there is a family history of convulsions. Another form of epilepsy, which may appear first during infancy, is due to organic disease of the cerebral cortex. The probable pathology of these cases is the sudden occlusion of a small surface vein by clot, with consequent congestion and hæmorrhagic softening of the part affected. The primary convulsion is usually of considerable duration and great severity, and occurs during an acute illness, or after exposure to some debilitating influence. In most cases only one side of the body is affected, and there may remain a certain amount of hemiplegia, usually slight, but extremely important from a diagnostic point of view. A third variety has been termed by the writer "birth palsy," as the initial lesion is meningeal hæmorrhage occurring at the time of birth, and caused by difficult or protracted labour. The treatment of infantile convulsions is discussed by Dr. Jules Simon[2] and Dr. Plicque,[3] who come to very similar conclusions. Attention is first directed to checking the seizure by the inhalation of chloroform or ether. The alimentary canal—the most common source of reflex disturbance—is to be relieved of its contents by emetics or enemata. Subsequently a rectal injection of musk and chloral hydrate will maintain the action of the chloroform, and, after the subsidence of all acute symptoms, bromide of potassium is to be administered by the mouth. Further examination must then be made as to the existence of other exciting causes, more especially uræmia, retention of urine, or a foreign body in the nose or ear, and suitable treatment adopted if one or other is present. A therapeutic measure found serviceable by Schumann[4] is the employment of abdominal massage, which in some cases of convulsions gives almost instantaneous relief.

**Infectious Fevers.**—Writing on the anomalies and complications of varicella, Dr. Galliard[5] says that the characteristic eruption of vesicles may be preceded, accompanied, or followed by a rash which is usually like that of scarlet fever. The vesicles may be confluent, or attain extraordinary dimensions (bullous or pemphigoid V.), or become pustular (globo-pustular V.). Hæmorrhage may take place from the vesicles, or from the nose, and even gangrene of these hæmorrhagic areas may supervene. A case of varicella bullosa is described by Mr. George Morgan,[6] who points out that this affection is sometimes erroneously termed acute contagious pemphigus. The existence of the latter disease is very doubtful, and if a case be admitted into hospital the usual sequence will probably be an outbreak of chicken pox.

In an editorial in the *Lancet*[7] regret is expressed that a difference of opinion exists on the subject of the notification of measles, considering that the death rate from that disease is from 6,000 to 14,000 per annum, and the sequelæ are frequent and serious. The difficulties in the way of notification are discussed, and the dual system is regarded as the only one likely to produce satisfactory results. The same subject is discussed in the *Practitioner*,[8] and the writer decides against a system of compulsory notification for the following reasons: (1) The infective period has been in existence for some time before the characteristic signs of the disease appear; (2) the statistics of those localities where it has been adopted do not show any manifest decrease in the death rate, and hence (3) the enormous expense necessary for carrying out notification loses its only justification; and (4) without isolation in hospital there is little hope of checking the spread of measles, and such isolation is not practicable. Antiseptic irrigation of the nose, mouth, and pharynx in measles has been employed by Dr. Siredey[9] with much benefit. In 50 cases of measles treated without irrigation, complications occurred in 23 (or 46 per cent.), while in 53 cases treated with irrigation, only seven (or 13 per cent.) were attended with complications. The lotions employed contained perchloride of mercury, or boracic acid, or permanganate of potash, or thymol and carbolic acid. While the results obtained are very satisfactory, the difficulties in carrying out the treatment as described by him will be found insuperable except in hospitals.

Dr. Ollivier[10] lays down the rule that nasal, buccal, and pharyngeal irrigations with antiseptic solutions should be employed as a means of disinfection after fevers, in addition to the other measures usually taken. The following are the periods of isolation he adopts: For scarlet fever, variola, and diphtheria, 40 days from the invasion; for measles and varicella, 16 days from the invasion; for pertussis, 21 days after the cessation of whooping; for mumps, 10 days after the disappearance of local symptoms. Dr. Clement Dukes[11] discusses at length the relations between school life and the spread of infectious disease, and urges strongly the necessity for providing "certificates of health" in the case of boys going from or to school at vacation times, when any infectious disease has existed at their homes or in the schools. The scarlatina-like rashes in children have proved a source of great perplexity to Dr. Henry Ashby,[12] and he refers to the various affections with which they are associated. In epidemic influenza a scarlatiniform rash is not uncommon, and when this is accompanied by other symptoms of scarlet fever the diagnosis is almost impossible in the individual case. Other affections in which similar rashes may appear are rubella, or epidemic roseola, acute suppuration, and diphtheria, while they are not unknown after large doses of belladonna.

**Respiratory Diseases.** — Dr. Donkin[13] says that asthma as it occurs during childhood is a neurosis with various exciting causes. In many cases there is a family history of asthma or of some other allied neurosis. He thinks that it is by no means a rare affection, but it is often regarded as bronchitis, pure and simple, and treated accordingly. This mistake is due to the fact that signs of catarrh of the bronchial tubes are usually present. Asthma often dates from an attack of measles or whooping cough. In the treatment of an acute attack he finds benefit from emetics, and in the more chronic cases he employs inhalations of nitre, and lobelia, and belladonna internally. At a meeting of the Harveian Society, Dr. L. G. Guthrie[14] showed two infants, aged three and six months, who were suffering from congenital respiratory spasm. The breathing was noisy, and was a combination of laryngeal stridor and pharyngeal stertor. The condition had been almost constant since birth, but varied in severity, being increased by sudden movements, draughts of air, bathing, or by any emotional disturbances. Cry and cough were natural, cyanosis was absent, and the dyspnœa caused no apparent distress. The tendency of such cases is towards recovery, and treatment does not appear to have any effect. As regards the etiology, he is inclined to agree with Dr. John Thomson in considering the condition as "a developmental neurosis," perhaps dependent on immaturity of the respiratory centres.

**Cardiac Diseases.**—In a paper on "The Heart in Chorea Minor," Professor Osler[15] draws the following conclusions: (1) That endocarditis is a very common complication of chorea minor. (2) That in the majority of such cases the endocarditis is independent of, and is not associated with, acute arthritis. (3) That in a considerable proportion of cases, much larger indeed than has hitherto been supposed, the complicating endocarditis lays the foundation of organic heart disease. These views coincide very closely with those of English physicians, who are inclined to regard chorea as a manifestation of the rheumatic diathesis in children, and both endocarditis and pericarditis as frequent accompaniments of chorea.

Dr. G. Variot[16] divides cases of congenital heart disease in children into those with, and those without, cyanosis. Cyanosis is of such importance that from it alone a diagnosis may be made, in the absence of other disease. Auscultatory signs are too untrustworthy and inconstant to admit of an exact determination of the cardiac condition. The existence of a congenital lesion with cyanosis, but without any murmur, is not very exceptional. Stenosis of the pulmonary artery alone, or with the addition of a patent foramen ovale, has frequently been found unaccompanied by cyanosis, but a perforation of the ventricular septum along with stenosis of the pulmonary artery is invariably accompanied by cyanosis.

[1] Clin. Jour., Sep. 5th, 1894, p. 309. [2] Gaz. des Hòpitaux, Feb., 1894, and N.Y. Med. Jour., July 14th, 1894, p. 43. [3] Gaz. Med. de Paris-March 24th, 1894, and Clin. Jour , June 6th, 1894. [4] Therap. Mon, atschr., March, 1894, and Ep. B.M.J., 2, 1894, p. 12. [5] La Med. Mod.. Jan. 13th, 1894, and N.Y. Med. Jour., June 2nd, 1894, p. 691. [6] B. M. J.. 2, 1894, p. 700. [7] May 26th, 1894, p. 1,313. [8] August, 1894, p. 148, [9] The Med. Week, August 17th, 1894. [10] Gaz. Med. de Strasb., No. 2, 1894, p. 17, and Am. Jour. Med. Sci.. Jan . 1894. [11] The Lancet, 2, 1894. p. 844. [12] Med. Chron., June, 1894. [13] Clin. Jour., Sep. 19th, 1894 [14] The Lancet, April 14th, 1894. [15] Med. Chron., Aug., 1894. [16] Jour. de clin. et du therap. infant., Ap. 12th, 1894, and N.Y. Med. Jour., May 5th, 1894.

